# Effect of the Peri-Annulated Dichalcogenide Bridge on the Bipolar Character of Naphthalimide Derivatives Used as Organic Electrode Materials

**DOI:** 10.3390/ma18092066

**Published:** 2025-04-30

**Authors:** Delyana Marinova, Lyuben Borislavov, Silva Stanchovska, Konstantin Konstantinov, Monika Mutovska, Stanimir Stoyanov, Yulian Zagranyarski, Yanislav Danchovski, Hristo Rasheev, Alia Tadjer, Radostina Stoyanova

**Affiliations:** 1 Institute of General and Inorganic Chemistry, Bulgarian Academy of Sciences, 1113 Sofia, Bulgaria; lborislavov@svr.igic.bas.bg (L.B.); stanchovska@svr.igic.bas.bg (S.S.); ydanchovski@chem.uni-sofia.bg (Y.D.); hrasheev@chem.uni-sofia.bg (H.R.); tadjer@chem.uni-sofia.bg (A.T.); radstoy@svr.igic.bas.bg (R.S.); 2 Faculty of Chemistry and Pharmacy, Sofia University “St. Kliment Ohridski”, 1164 Sofia, Bulgaria; k.konstantinov@pharmfac.mu-sofia.bg (K.K.); ohmgm@chem.uni-sofia.bg (M.M.); ohss@chem.uni-sofia.bg (S.S.); ohjz@chem.uni-sofia.bg (Y.Z.); 3 Faculty of Pharmacy, Medical University of Sofia, 1000 Sofia, Bulgaria

**Keywords:** organic electrode materials, *n*- and *p*-type redox reactions, 1,8-naphthalimide derivatives, *peri*-dichalcogenide bridge

## Abstract

In recent years, bipolar organic electrode materials have gained recognition as competitive alternatives to inorganic materials due to their unique multielectron redox mechanism for energy storage. In this study, we examined the mechanism of redox reactions in naphthalimide (NI) derivatives when used as electrodes in lithium half-cells with ionic liquid electrolytes. The NI derivatives consist of three building fragments: an aromatic naphthalene core, *N*-alkylated imide unit, and a *peri*-dichalcogenide bridge. The integration of electrochemical and microscopic methods with DFT calculations facilitates the delineation of the role of each fragment in the oxidation and reduction reactions of NI derivatives. It is found that the *peri*-dichalcogenide bridge is mainly involved in the oxidation of NI derivatives above 3.9 V, the charge compensation being achieved by electrolyte TFSI^−^ counter-ions. The reduction of NI derivatives with two Li^+^ ions is mainly due to the participation of the chalcogenide bridge, while after interaction with the next two Li^+^ ions, the imide fragment and the naphthalene moiety contribute equally to the reduction. Based on the leading role of the *peri*-dichalcogenide bridge, the redox properties of NI derivatives are effectively controlled by the gradual replacement of S with Se and Te atoms in the bridge. To improve the electronic conductivity of NIs, composites with rGO are also synthesized by a simple procedure of mechanical mixing in a centrifugal mixer. The composites rGO/NIs display a good storage performance, the best being the Se-containing analogue.

## 1. Introduction

Organic electrode materials (OEMs) have recently been considered as competitive alternatives to inorganic electrode materials because they amalgamate high energy density with low cost and environmental friendliness, as well as a wide variety of compositions and architectures with the ability to withstand the structural stress that occurs during redox reactions [1,2,3]. The OEMs store energy by means of three different types of redox mechanisms: (i) OEMs are easily reduced during the interaction with alkali or polyvalent ions (*n*-type); (ii) OEMs undergo oxidation, the positive charges being compensated by the counter anions from the electrolyte (such as TFSI^−^, FSI^−^, PF_6_^−^) (*p*-type); (iii) OEMs composed of *n*- and *p*-type units can undergo simultaneous oxidation and reduction with the participation of both cation and anion counterparts from the electrolyte (bipolar type) [4,5,6]. While *n*-type OEMs have higher capacities and slow kinetics, *p*-type OEMs are characterized by faster kinetics and a higher redox potential [2,4]. The bipolar OEMs unify the specific features of *n-* and *p*-type OEMs, by avoiding their disadvantages [3,6].

Naphthalimide (NI) derivatives deliver high capacity combined with ecological benefits, making them attractive electrodes for lithium-ion batteries (LIBs) [7]. The main representative is the 1,4,5,8-naphthalene diimide, which serves as a model *n*-type redox molecule with an average potential of 2.5 V and capacity of around 176 mAh/g [8]. Given the high electron affinity of NI derivatives, the simple synthetic strategy to modulate both the redox properties and cycling stability is by appropriate donor substitutions [9,10]. The next strategy to improve the redox properties of NIs is to polymerize them. Instead of naphthalene diimide, polymerization into three-dimensional polyimide frameworks enables the creation of effective cathodes for LIBs, characterized by excellent long-term stability and reversibility in cycling [11]. The formation of a two-dimensional polymer containing unique π-conjugated structural unit has been shown to exhibit an improved diffusion of ions inside the 2D molecular channels [12]. The combination of *n*-type NIs with *p*-type moieties in a bipolar molecule is an effective way of controlling the electrochemical performance of OEMs. The *n*-*p*-*n* molecular architecture comprising *N*,*N*’-bis (2-anthraquinone)-dihydrophenazine with a theoretical capacity of 270 mAh/g has recently been designed as a bipolar electrode material for Li-ion batteries [13]. By polymerizing NIs, ambipolar triphenylamine-based polynaphthalimides (TPA-PNIs) have been reported that can simultaneously act as a cathode, anode, and binder in LIBs [14]. The association of an *n*-type naphthalene diimide unit with two *p*-type viologen units leads to the formation of a multielectron electrode with a high specific capacity close to the theoretical one [15]. Recently, we have proposed a new molecular architecture for bipolar OEMs [16,17]. The molecular architecture is based on the synergetic coupling of the naphthalimide core with the *peri*-diselenide or disulfide bridge. The redox properties and the mechanical stability of the naphthalimide core are further modified by introducing halogen atoms at positions 3 and 6 and by varying the alkyl chain from 4 to 8 [16,17].

Despite the progress in OEMs, key challenges remain, namely the potential dissolution in electrolytes and low electronic conductivity of OEMs [1,2,3]. In this respect, molecular engineering plays an essential role in controlling the solubility of NI derivatives in compliance with the battery type. While electrode solubility is a drawback for rechargeable batteries, it is a merit for flow batteries. Thus, substituents of higher steric volume at the nitrogen in the naphthalimide core (e.g., *N*-butylnaphthalimide) improve their solubility in non-aqueous electrolytes, as a result of which *N*-substituted naphthalimides have been employed as a stable anolyte material for non-aqueous redox flow batteries [18]. Carbon coating of *N*,*N*’-bis (2-anthraquinone)-dihydrophenazine delays its dissolution and dramatically improves its electrochemical performance in lithium electrolyte containing 3M LiFSI in TEGDME [13]. The dissolution of the NI derivatives is a function of choosing the appropriate electrolyte [19]. A better electrochemical performance of naphthalene diimide is observed when ether-based electrolytes (such as 1M LiTFSI or 1M LiClO_4_ in dioxolane/dimethoxyethane) are used instead of the usual carbonate-based electrolytes (such as 1M LiTFSI or 1M LiPF_6_ in EC/DEC) [8]. A highly concentrated electrolyte containing NaTFSI in acetonitrile (molar ratio of 1:2.7) is effective in suppressing the dissolution of the high-voltage sodium croconate electrode, thereby enhancing its rate capability [20]. In addition, the ionic liquids act as suitable electrolytes to reduce the solubility of NIs and boost their electrode performance [16,17].

Another factor affecting OEM redox properties is electrical conductivity [21]. There are several strategies to improve the electrical conductivity of OEMs: (i) to produce OEM thin films or to reduce the dimensionality to the nanoscale to make it comparable to the diffusion length of charge carriers [21,22]; (ii) to fabricate polymers of OEMs with intrinsic porosity [21,23]; (iii) to make composites of OEMs with conductive carbonaceous materials [24,25]. Among the various strategies, the simplest is to bond OEMs to conductive carbons by adsorption or polymerization methods [21]. As conductive carbons, carbon fibers, CNTs, graphene, rGO, or porous carbons are the most commonly used additives because they allow the formation of a conductive network, which facilitates electron transport inside the electrode and maximizes its performance [21,26,27]. Furthermore, the advantage of using CNTs relies on the formation of π-π interactions between CNTs and OEMs. This has been well demonstrated by Wang et al. [28], who succeeded in attaching CNTs to poly (TEMPO methacrylate, PTMA) via π-π interactions between them. A thus-obtained composite displayed 27% improvement in capacity and excellent performance under a high rate of 5500 mA/g [28]. The introduction of graphene into PTMA via a dispersing-deposition technique enables high rate capability and cycling stability to be achieved for organic electrodes, as evidenced by a high reversible capacity of 226 mAh/g at 0.1 C and approximately 80 mAh/g at an ultra-high rate of 200 C [29]. Due to the difficulties in synthesizing graphene, most research has focused on replacing graphene with the cheaper and easier to prepare reduced graphene oxide (rGO) as a component in composites with OEMs [30]. Composites of a porous polymer with rGO provide high specific capacity, excellent cycling stability, and reversibility in sodium ion cells due to the special porous structure of the polymer and its enhanced electronic conductivity induced by the introduction of rGO [31]. This composite performs better in the ether-based electrolyte than in the conventional ester-based electrolyte [31].

Exploiting the redox properties of NI derivatives, the preparation of composites with carbonaceous additives allows us to improve their electrochemical performance as electrodes in lithium-ion cells. In this study, we select a *peri*-dichalcogenide bridge annulated to a naphthalimide core as a bipolar redox molecular architecture. This architecture facilitates the progressive variation in the chalcogenide atoms in the bridge from S to Se and Te, and consequently, the effect of chalcogenides with decreasing electronegativity on the ability of NI derivatives to participate in both oxidation and reduction reactions can be precisely evaluated. All compounds contain chlorine atoms at positions 3 and 6, and the alkyl chain is fixed at C8. The redox properties of NI derivatives are monitored by using them as electrodes in lithium-ion cells with an electrolyte based on ionic liquids. The experimental findings are rationalized in the framework of DFT calculations. To improve the electronic conductivity of NIs, composites with rGO are also synthesized by a simple procedure of mechanical mixing in a centrifugal mixer.

## 2. Materials and Methods

All starting materials (except those synthesized by us) and solvents were commercially available and used without additional purification (Merck, Fisher Scientific, Fluorochem, Sigma-Aldrich, St. Louis, MO, USA). Thin-layer chromatography was used to monitor the progress of all reactions (Macherey-Nagel F 254 silica gel sheet, Macherey-Nagel, Duren, Germany) using appropriate mixture of solvents as eluent (described for each compound in the synthetic procedures). Column chromatography on silica gel (Macherey Nagel, 0.063–0.200 mm) was used for purification. NMR spectra were recorded on a Bruker Avance 500 MHz instrument (Bruker, Karlsruhe, Germany). Spectra (1H, 13C{1H}) were referenced to appropriate residual solvent signals (CDCl_3_, C_2_D_2_Cl_4_). Elemental analyses were carried out on a Leco CHNS-932 (Leco Europe, Geleen, The Netherlands).

### 2.1. Synthesis

3,9-dichloro-6-octyl-5H-[1,2]dithiolo [3′,4′,5′:4,5]naphtho [1,8-cd]pyridine-5,7 (6H)-dione (**SCl8**) and 3,9-dichloro-6-octyl-5H-[1,2]diselenolo [3′,4′,5′:4,5]naphtho [1,8-cd]pyridine-5,7 (6H)-dione (**SeCl8**) were prepared according to the procedures previously reported by us [16,17].

Synthesis of 3,9-dichloro-6-octyl-5H-[1,2]ditellurolo [3′,4′,5′:4,5]naphtho [1,8-cd]pyridine-5,7 (6H)-dione (**TeCl8**).

Te (9 mmol) and Na (10.5 mmol) were dissolved in 30 mL of degassed NMP. The suspension was heated to 145 °C under a double Ar balloon for 90 min. During this time, the suspension turned dark purple, indicating the formation of Na_2_Te_2_. The solution was then cooled to room temperature.

**Scheme 1:**
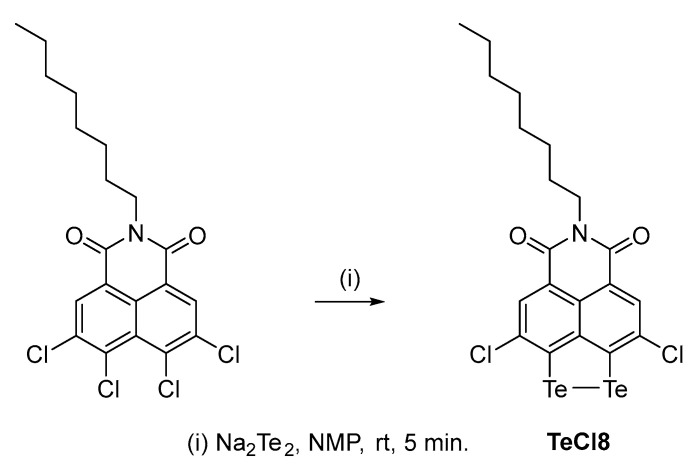
Synthesis of TeCl8.

In a separate flask, 1.34 g (3 mmol) of *N*-octyl-3,4,5,6-tetrachloronaphthalimide was dissolved in degassed NMP (0.1 M). The cooled solution of the tellurium salt was added to the flask using a syringe. After 5 min, 60 mL of THF was added to the solution, and the resulting suspension was filtered using a Celite plug. Acidified distilled water was then added to the filtrate, instantly precipitating the product (Scheme 1). After 15 min, the solid was filtered through a glass-fritted filter and continuously washed with methanol to remove residual NMP and THF. The product was vacuum-dried for 5 h. Overall yield: 1.14 g (60%).

**^1^H NMR** (δ (ppm), 500 MHz, 1,1,2,2-Tetrachloroethane-*d*_2_): 8.31 (s, 2H), 4.12 (t, J = 7.6 Hz, 2H), 1.69 (m, 2H), 1.22–1.44 (m, 12H),0.88 (t, J = 7.2 Hz, 3H) (see Figure S1A).

**^13^C NMR** (δ (ppm), 126 MHz, 1,1,2,2-Tetrachloroethane-*d*_2_): 162.14, 144.36, 140.67, 134.89, 129.96, 129.38, 122.96, 40.71, 31.69, 29.22, 29.11, 27.84, 27.00, 22.56, 14.11 (see Figure S1B).

**Elemental analysis**: calc. for C_20_H_19_Cl_2_NO_2_Te_2_: %C 38.04, %H 3.03, %N 2.22; found: %C 38.12, %H 3.11, %N 2.18.

For convenience, the samples containing S, Se, and Te are further referred to as **SCl8**, **SeCl8**, and **TeCl8**.

### 2.2. Preparation of Composites of NI Derivatives with rGO and Electrode Fabrication

The composites of naphthalimide derivatives (**SCl8**, **SeCl8**, **TeCl8**) and rGO were obtained by mixing them in a ratio of 85 wt% to 15 wt%, respectively. The weighed amounts were first vigorously ground in an agate mortar, then transferred to a planetary mixer (Thinky ARE250, Tokyo, Japan) and homogenized for 30 min with a small amount of alcohol.

### 2.3. Characterization Techniques

Swagelok-type two- and three-electrode model cells were used for testing. The electrodes were prepared by mixing the active materials (i.e., NI derivatives or composites) with a conductive carbon black (C65) additive and a carboxymethylcellulose (CMC) bonding additive in a ratio of 70–20–10 wt%. CMC is a less-air-sensitive water-based binder, which, in our case, results in better collector adhesion. The slurry prepared in a planetary mixer was spread on carbon-coated aluminum foil and dried under vacuum at 120 °C overnight. Discs of suitable diameter were cut out and dried again under vacuum before assembly, with an active mass of approximately 2–3 mg. The electrolyte was a 1 M solution of (LiTFSI-Pyr_1,3_FSI (lithium bis (trifluoromethanesulfonyl) imide) in *N*-methyl, propyl (pyrrolidinium bis (fluorosulfonyl) imide), 1:9 by ratio). An MB-Unilab glovebox model Pro SP (1500/780) (M. BRAUN INERTGAS-SYSTEME GmbH, D-85748 Garching, Germany) was used for an ultra-dry, oxygen-free environment for assembling the model cells. The electrochemical tests in potentiostatic and galvanostatic mode were performed on a multi-channel potentiostat/galvanostat Biologic VMP-3e (BioLogic, 38170 Seyssinet-Pariset, Grenoble, France), and eight-channel system Arbin LBT2000 (Arbin Instruments, College Station, TX 77845, USA).

The scanning electron microscopy and EDS analysis were carried out on JEOL JSM 6390 (JEOL Ltd., Tokyo, Japan), equipped with an AZtec Oxford Instruments 6.0 energy dispersive X-ray spectrometer (Oxford Instruments Croup, High Wycombe, UK) to observe the morphology and determine the chemical composition of the *peri*-substituted dichalcogenides.

The color photographs were taken on a Raman microscope with a digital camera for observing the sample with a spatial resolution of XY ≤ 0.5 µm and Z ≤ 2.0 µm, and an objective with a working distance of 100× (InVia Qontor Raman Confocal Microscope, Renishaw plc, Wotton-under-Edge, UK).

### 2.4. Computational Protocol

The quantum chemical calculations were performed with the hybrid DFT functional B3LYP [32,33] utilizing the 6-31+G* basis set [34,35] for all atoms except for Te, for which def2-SVPD was applied [36]. Model Cl-derivatives of NIs with chalcogenide bridges, in which the alkyl tail (C8) is limited to a –CH_3_ group are constructed and labelled SCl, SeCl, and TeCl. The charge of the oxidation products is compensated by an equivalent number of explicit counter-ions and the entire system is modelled in implicit solvent. The latter is simulated with the PCM scheme [37,38,39] with ε = 15 for the ionic liquid. Dispersion is accounted for implementing the D3-BJ empirical correction [40,41]. The population analysis used the NBO atomic charges [42,43]. All calculations were carried out with the Gaussian 16 software package [44].

## 3. Results and Discussions

The proposed synthetic procedures for obtaining the target dichalcogenides demonstrate excellent results on the gram scale employed. Furthermore, the following considerations lead us to believe that they can be readily adapted to larger scales for potential industrial application. The starting tetrachloronaphthalimide is obtained in quantitative yield from readily available reagents. The work-up and purification in the dichalcogenation step involve only filtration and washing, obviating the need for chromatographic columns or other expensive techniques. All solvents and reagents, including sulfur, selenium, and even tellurium, are available at competitive prices.

### 3.1. Electrochemical Redox Reactions at **SCl8**, **SeCl8**, and **TeCl8** Electrodes

**Figure 1:**
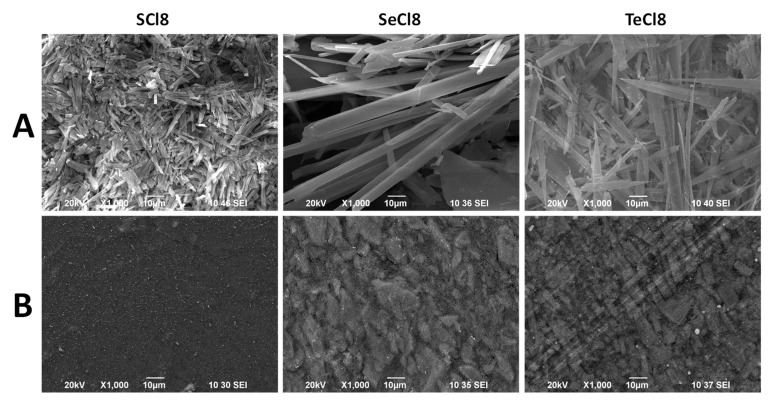
SEM images of **SCl8**, **SeCl8**, and **TeCl8** (**A**) and the electrodes (**B**) fabricated from them.

Prior to electrochemical characterization, the first step is to fabricate electrodes from organic compounds with a relatively smooth surface [45]. Figure 1 compares the morphology of pristine **SCl8**, **SeCl8**, and **TeCl8** and the electrodes fabricated from them. The morphology of **SCl8** consists of small micrometric sticks that are tightly bound together. The sticks have a length of about 10 μm and a cross section of about 1 μm (Figure S2). Replacing S with Se produces well-faceted rods with a length of about 100 μm and a cross section of about 2–5 μm. At the end of the rods, small, faceted particles can be seen, with sizes of around 1–2 μm (Figure S2). Further replacement of Se with Te results in the formation of wire-like particles with needle-like ends (Figure S2). Irrespective of the different morphology, all the electrodes made from **SCl8**, **SeCl8**, and **TeCl8** have a satisfactory smooth surface (Figure 1B). This indicates that the compounds are well mixed with the activated carbon additives and the CMC binder to form robust networks within the electrodes.

**Figure 2:**
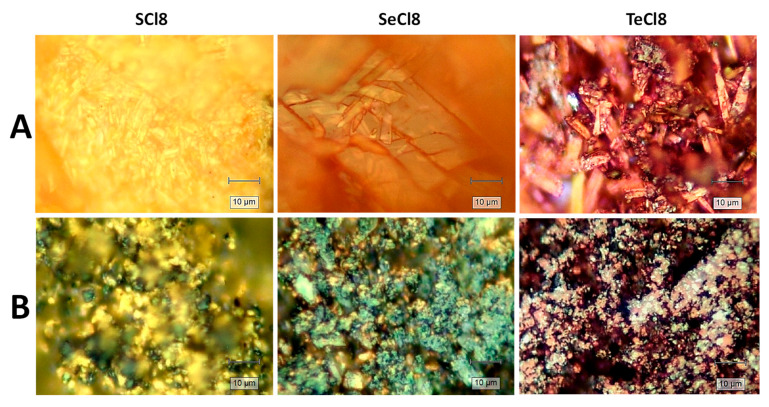
Colour images of **SCl8**, **SeCl8**, and **TeCl8** (**A**) and their composites with rGO (**B**).

Given the intrinsically low electronic conductivity of organic compounds, composites of NIs with rGO are prepared by a mechanical mixing in a centrifugal mixer. The effectiveness of mixing is monitored by a Raman microscope (Figure 2). For the pristine compositions, the Raman images reveal the main SEM features: going from **SCl8**, to **SeCl8**, and to **TeCl8**, the morphology changes progressively from small tightly bound sticks, to rod-like particles, and to wire-like particles with needle-like ends. The images also display the changing colors of **SCl8**, **SeCl8**, and **TeCl8**, which are yellow, orange, and violet, respectively. For all samples, mixing with rGO causes the pristine particles to break down into small components that adhere to each other in spherical aggregates aided by rGO (Figure 2B). This proves that rGO and NIs are well mixed even after using a simple mixing method.

**Figure 3:**
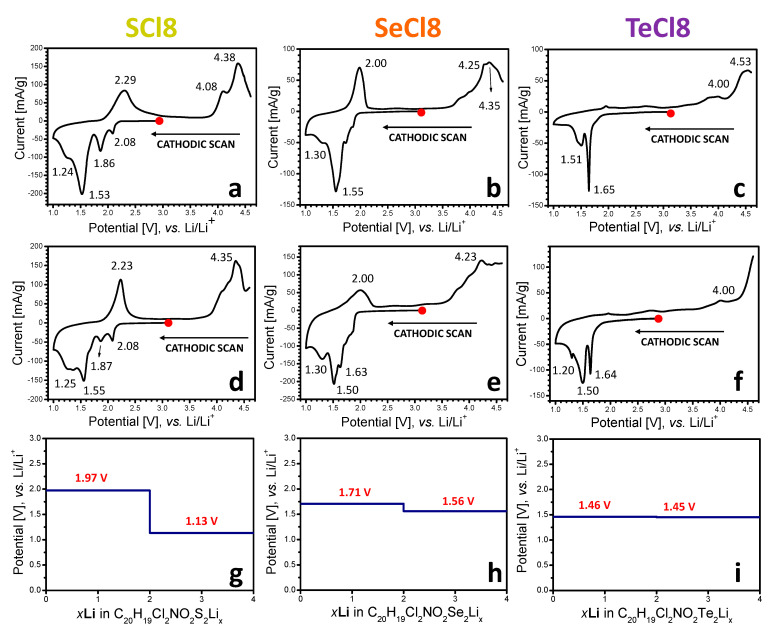
CV curves of **SCl8** (**a**), **SeCl8** (**b**), and **TeCl8** (**c**) and their composites with rGO: **SCl8/rGO** (**d**), **SeCl8/rGO** (**e**), and **TeCl8/rGO** (**f**), after the first cathodic scan, followed by the anodic scan. The calculated potential profiles of stepwise reduction of the respective model compounds **SCl** (**g**), **SeCl** (**h**), and **TeCl** (**i**) with Li.

**Figure 4:**
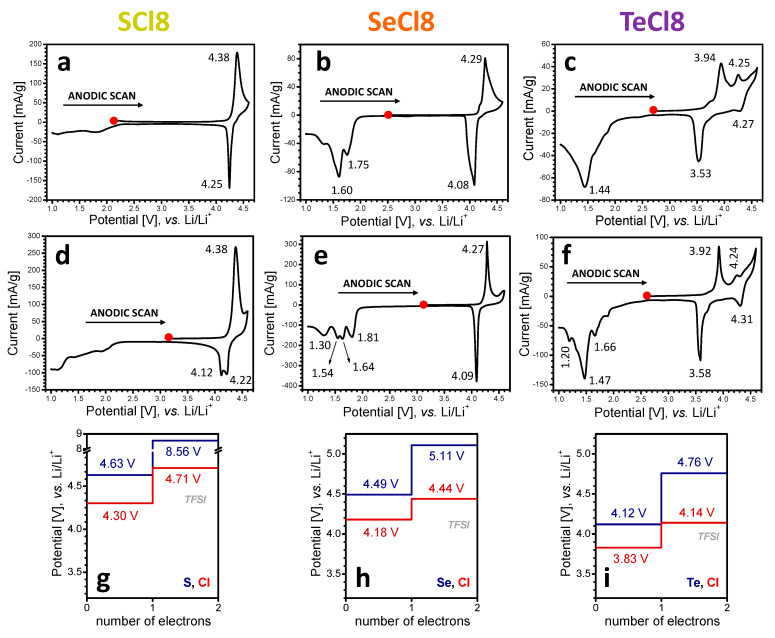
CV curves of **SCl8** (**a**), **SeCl8** (**b**), and **TeCl8** (**c**) and their composites with rGO **SCl8/rGO** (**d**), **SeCl8/rGO** (**e**), and **TeCl8/rGO** (**f**), after the first anodic scan, followed by the cathodic scan. The calculated potential profiles of stepwise oxidation of the respective model compounds **SCl** (**g**), **SeCl** (**h**), and **TeCl** (**i**) without (blue) and with (red) account of the counter-ion (TFSI^−^).

The redox reactions at **SCl8, SeCl8**, and **TeCl8** are monitored by means of CV experiments (Figure 3). To differentiate between the reduction and oxidation of NIs, Figure 3 and Figure 4 compare the CV curves starting from the first cathodic and anodic scans, respectively. After the first cathodic scan of **SCl8**, several superimposed reduction peaks can be distinguished, namely at 2.08 V, 1.86 V, 1.53 V, and around 1.24 V (Figure 3a). Compared to **SCl8**, the CV curve of **SeCl8** displays reduction peaks at 1.55 V and 1.30 V, together with shoulders between 1.8 and 1.7 V (Figure 3b). For **TeCl8**, reduction peaks at 1.65 V and 1.51 V become visible (Figure 3c). This indicates that the reduction potentials are distributed over a range whose limits decrease successively from S to Se and to Te, in accordance with their decreasing electronegativities. The same trends in alteration of the reduction potentials and potential range are observed for the composites consisting of NI derivatives and rGO (Figure 3d–f). Compared to the net compounds, the CV curves of the composites show the same positions of the reduction peaks, but with a better resolution. The close inspection of the CV curve profiles indicates that both the chalcogenide element and the presence of rGO determine the current density. This means that the samples deliver a different capacitance after the first reduction. The reduction capacitance calculated from the CV curves is listed in Table S1 (for the sake of comparison, the data are calculated as specific capacity). The comparison shows that the specific capacity decreases progressively with the electronegativity of the chalcogenide element. After the addition of rGO, the specific capacity increases for all samples, especially for the Se-containing sample. Thus, in the composites, the specific capacity of **SeCl8** becomes slightly higher than that of **SCl8**, while the specific capacity of **TeCl8** remains lower. The variation in the specific capacity of the samples can be related to the electrical conductivity of the electrodes: the higher the capacity, the more conductive the electrodes. Thus, the enhancement of the specific capacity after rGO addition is a simple consequence of the high electron conductivity of rGO [21], while for the net compositions, the different specific capacities suggest a dependence of their electron conductivity on the chalcogenide element. Among the NI derivatives, the rGO component appears to have a better contact with the SeCl8, which is reflected in the higher specific capacity. This deserves further investigation. In addition, the dependence of the reduction capacity on the presence of rGO can explain the observed small changes in the ratio between the reduction peaks, especially those at 2.08 and 1.86 V for **SCl8** (Figure 3a,d). Importantly, the CV curves for net compounds and rGO composites display close profiles in terms of the peak positions, demonstrating that the reduction is an intrinsic property of the NI derivatives. During the reverse anodic scan, peaks at 2.3 and 2.0 V appear for **SCl8** and **SeCl8**, respectively, while for **TeCl8** the reverse peak is hardly visible.

After the first anodic scan, the CV curves of all compounds display an oxidation peak, whose position decreases in line with the chalcogenide electronegativity: the oxidation potential was determined to be 4.38 V for **SCl8** (Figure 4a), 4.29 V for **SeCl8** (Figure 4b), and 3.94 V for **TeCl8** (Figure 4c). In addition, **TeCl8** also shows an extra oxidation peak at 4.25 V. The comparison of the first reduction and oxidation of NIs clearly demonstrates that the nature of the chalcogenide atoms in the bridge exerts a significant influence on the oxidation potential, and, to a lesser extent, on the reduction potential. Furthermore, the reversibility of the oxidation reaction is better than that of the reduction. This reflects the different mechanisms of NI reduction and oxidation. As in the case of reduction, the formation of composites between NIs and rGO does not affect the CV curve profiles in terms of the position of the oxidation peak and its dependence on the nature of the chalcogenide atoms (Figure 4d–f). Furthermore, the current density of the composites is significantly higher than that of the net compounds, thus indicating an increase in the oxidation capacity after the addition of rGO (Table S1). This is the next evidence of the improved electrical conductivity of the composite electrodes.

In general, the studied NI derivatives are reduced and oxidized in very different potential windows, i.e., below 2.0 V and above 4.0 V, respectively. Although the reduction and oxidation processes exhibit different reversibility, the CV curves become almost identical after five scans of the cell, regardless of whether anodic or cathodic scans are started first (Figure S3).

### 3.2. Mechanism of the Redox Reactions in **SCl8**, **SeCl8**, and **TeCl8**

**Figure 5:**
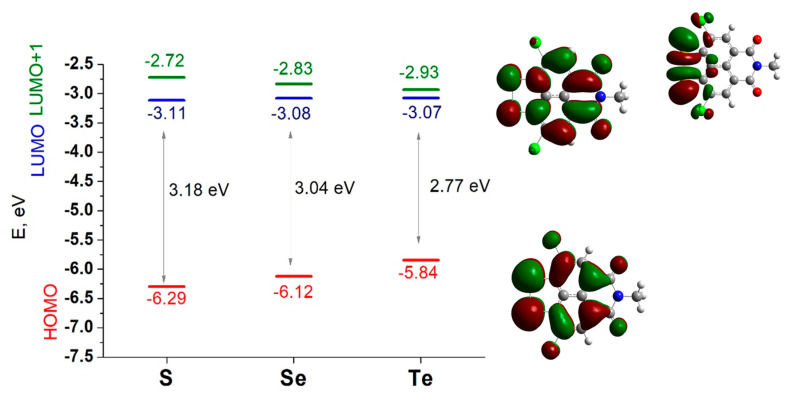
Energy (in eV) of the highest occupied (HOMO) and the two lowest unoccupied (LUMO and LUMO+1) molecular orbitals of the modeled S-, Se-, and Te-containing NIs.

To rationalize the oxidation and reduction of NIs, DFT calculations on model systems are performed. Figure 5 presents the energy and images of the canonical frontier orbitals of the three models. The shapes of HOMO and LUMO of the three compounds are identical—all of them are of π-type, asymmetric with respect to the c_2_ axis, and delocalized over the molecular periphery, excluding the atoms lying on the axis. The latter signifies that the alkane chain (C_8_) will have no impact on the redox properties of the compounds and its curbing to -CH_3_ in the models is judicious [16,17].

The HOMO-LUMO gap decreases from SCl to TeCl, which is an inference for conductivity enhancement of the compounds in the same order.

The E (LUMO) values vary within a narrow energy range of 0.04 eV, all of them negative. This signals that the molecules would accept electrons with a reduction potential slightly dependent on the chalcogenide atoms. However, very close in energy to the LUMO, is LUMO+1, which is of the σ_p_ type, localized on the chalcogenide bridge. The energy difference between the two frontier antibonding MOs gradually decreases from SCl (0.39 eV) to SeCl (0.28 eV) and TeCl (0.14 eV), which is an implication that the potential range upon reduction progress will become narrower. This is in good agreement with experimental observations made on the relationships between chalcogenide atoms and the potential reduction range (Figure 3). Compared to E (LUMO), E (HOMO) grows steadily from SCl to TeCl (Figure 5), indicating a decrease in the oxidation potential in this order, as observed after the first anodic scan (Figure 4). The MO shape implies active participation of the chalcogenide bridge in the oxidation as well.

**Figure 6:**
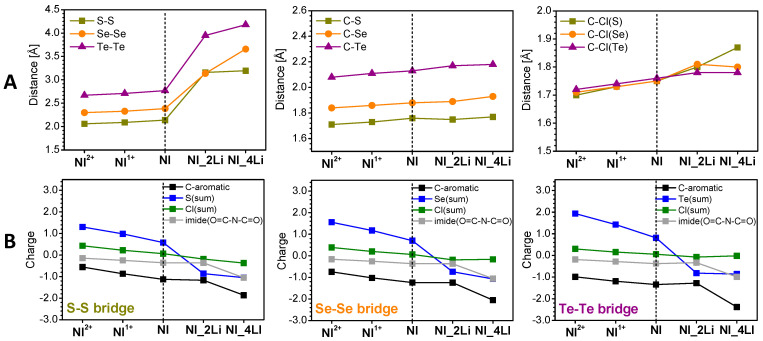
Calculated averaged lengths (in Å) of key bonds (**A**) and charge distribution among the different molecular fragments (**B**) of oxidized (2+, 1+), neutral, and reduced (2Li, 4Li) SCl, SeCl, and TeCl. The numerical values can be found in Tables S2 and S3.

The reduction was modeled by placing Li atoms at all viable locations and choosing the lowest-energy one as a representative of the respective degree of lithiation. Two Li atoms are added at each step to maintain the system closed-shell. There are two positions that are the most attractive for the first two lithium atoms for all compounds: either close to the chalcogenide bridge or close to the oxygens of the nitride fragment (Figure S4). The energy difference between the two options is quite large for SCl, decreases for SeCl, and becomes small for TeCl. Thus, for SCl_2Li and SeCl_2Li, the preferred structures are when Li^+^ interacts with the chalcogenides, while for TeCl_2Li both options are feasible. For all three models, the distance between the chalcogenides in the bridge (Figure 6A left, Figure S4, Table S2) increases dramatically (more than 40%) in this first reduction step. This is supported by the calculated NBO charges (Figure 6B, Table S3, Figure S5), which disclose that in the first reduction step (2Li) 70–80% of the negative charge is localized at the chalcogenide bridge. It is important to note that despite the enormous increase in the distance between the chalcogenides, the C-S/Se/Te bond is close to that of the neat molecules. At the same time, the C-Cl bond undergoes changes, but not as significant as the chalcogenide bonds (less than 2%). This means that the chalcogenides and chlorine remain bound in the compounds lithiated up to 2Li.

The introduction of the next two Li atoms results in structures, which are a hybrid of the two options for 2Li accommodation. SCl retains the symmetry upon reduction, while SeCl and TeCl undergo deformations (Figure S4). The C-S/Se/Te bonds continue to increase, but not as significantly as after the first reduction step (Figure 6, Table S2). Overall, the remaining structural changes (i.e., C-S/Se/Te and C-Cl bonds) are very small. The NBO charges (Table S3, Figure S5) evidence that the lithiation with 4Li has a lesser effect on the chalcogenide bridge compared to the reduction with 2Li. In this case, the imide fragment comes into play together with the entire naphthalene unit with equal contribution of these two fragments to the charge reallocation. The chlorines take practically no part in the charge redistribution during reduction.

However, the next lithiation steps (i.e., 6Li in total) result in some destructive reactions, in which the lithium ions abstract chlorine from the compounds (Figure S6). This may lead to two consequences: (i) chlorine deficiency and (ii) degenerative side reactions. Both of them damage the electrodes with a negative impact on the cycling stability. Thus, the calculations clearly demonstrate that the molecular architecture of NI derivatives is stable under reduction with a total of four Li, whereas degradation processes occur upon interaction with the next two Li atoms.

As a measure of the comparison between the calculated and experimental data, Figure 3g–i gives the calculated standard potentials of the reduction stages of the three compounds. In general, all reduction potentials fall in the potential range between 1 and 2 V vs. Li/Li^+^, which is in perfect agreement with the experimental observations (Figure 3a–f). If the first two peaks in the CV curve in the cathodic scan of **SCl8** and **SeCl8** correspond to the consecutive reduction by two electrons (or two Li^+^, Figure 3a,b), the computed values are exactly their average (Figure 3g,h). The difference between the potentials corresponding to the reduction with two and four Li atoms diminishes in the order S > Se > Te, culminating in almost-indistinguishable two-stage potentials at TeCl (Figure 3i). This is the experimental order that we observed by the first cathodic scan of NI derivatives, too (Figure 3c,f).

Compared to reduction, the oxidation of NIs proceeds via the formation of cations, whose charges are compensated by the counter-ions from the electrolyte (i.e., TFSI^−^). The inclusion of counter-ions in the calculation of the standard potentials leads to a systematic lowering of the potential and thus to a better approximation to the experimental data (Figure 4). The calculated first and second oxidation potentials decrease gradually with chalcogenide atoms in the order S > Se > Te, corresponding to the HOMOs alignment (Figure 5). This trend is maintained regardless of the presence or absence of the TFSI^−^ counter-ions. Importantly, the calculated oxidation potentials in the presence of TFSI^−^ are consistent with the experimentally determined ones: 4.38 V vs. 4.30 V for **SCl8**, 4.27 V vs. 4.18 V for **SeCl8**, and 3.83 V vs. 3.92 V for **TeCl8**, respectively. Only for **TeCl8**, the two oxidation peaks observed in the CV curve are well reproduced by the calculated oxidation potentials in the presence of TFSI^−^.

The decrease in oxidation potentials in the order S > Se > Te follows the charge localization in the oxidized NI molecules (Figure 6, Table S3, Figure S5). To quantify the charge localization, the changes in the charge of different molecular fragments are calculated and listed in Table S3. The charge transfer between the ions and counter-ions grows in the order S < Se < Te. As one can see, the localized charge follows the same order S < Se < Te in the percentage of the acquired charge 40/50/60, respectively. The naphthalene carbons are involved most markedly in SCl and negligibly in TeCl. It is remarkable that the NBO charges of Cl are positive even in the pristine molecule. The Cl substituents also acquire a share of the positive charge, but in the opposite order: Cl(S) > Cl(Se) > Cl(Te). In the same order and with a similar share is the imide chain (O=C-N-C=O) contribution. Upon oxidation, it is visible that there is a slight contraction within ≤0.1 Å of all key bonds (Figure 6, Table S2), indicating good stability of the molecular structure during oxidation.

Given the good agreement between the experimental and calculated potentials, it can be assumed that the calculation models using even just one molecule are sufficiently adequate in describing the reduction and oxidation processes. The next question is why the chalcogenide bridge has a different role in the oxidized and reduced forms. A comprehensive interpretation of this phenomenon can be given from an electronic standpoint, particularly from a π-electronic perspective. Let us take a look at the molecular architecture: it consists of an aromatic naphthalene unit with undisturbed by the Cl-substituents delocalization, an imide chain (O=C-N-C=O), and a chalcogenide bridge (A-A, A=S, Se, Te). The latter two are bound to the naphthalene unit to form heterocycles. Each heterocycle contains seven π-electrons and is thus non-aromatic. To achieve aromatic stability (i.e., six π-electrons, according to Hückel’s (4n + 2)-rule), each of them is prone to losing an electron. The exocyclic oxygens withdraw electron density from the N-heterocycle and aromatize it, but nothing stabilizes the A-heterocycle, as the naphthalene unit is not ‘cooperative’ in adopting more electron density. Thus, oxidation is a favorable process, aromatizing the A-cycle as well, the largest share of positive charge belonging to the A-atoms, with the share growing in the order of the atomic numbers. The second oxidation, however, is less favorable, since it again disturbs the aromaticity. This explains the high values of the second oxidation potential and the role of the explicit counter-ions, which not only polarize the NIs (as does the implicit solvent) but also contribute some amount of compensatory electron density, which results in substantial depression of the calculated second oxidation potential: 3.85 V, 0.67 V, and 0.52 V for (SCl)^2+^, (SeCl)^2+^, and (TeCl)^2+^ compared to 0.33 V, 0.31 V, and 0.29 V for (SCl)^+^, (SeCl)^+^, and (TeCl)^+^, respectively.

While oxidation stabilizes the A-cycle, reduction leads to an antiaromatic 8-π-electronic structure. Antiaromatic cycles are aromatized by adopting two electrons to comply with Hückel’s rule, which explains the most favorable localization of the first two Li atoms in the proximity of the A-A bridge. The cleavage of the A-A bond (Figure S4) is invoked by the presence of Cl. The other stabilization avenue for antiaromatics is the substantial geometry change, deplanarization of the cycle being the preferred choice. This explains the drastic deformation in SeCl_4Li, TeCl_2Li, and TeCl_4Li (Figure S4), where the two A-atoms are bridged by Li, thus forming an 8-π-electron non-planar structure. This also explains the drastic change in the A-A distance upon reduction in all three compounds (Table S2).

### 3.3. Storage Performance of **SCl8**, **SeCl8**, and **TeCl8**

**Figure 7:**
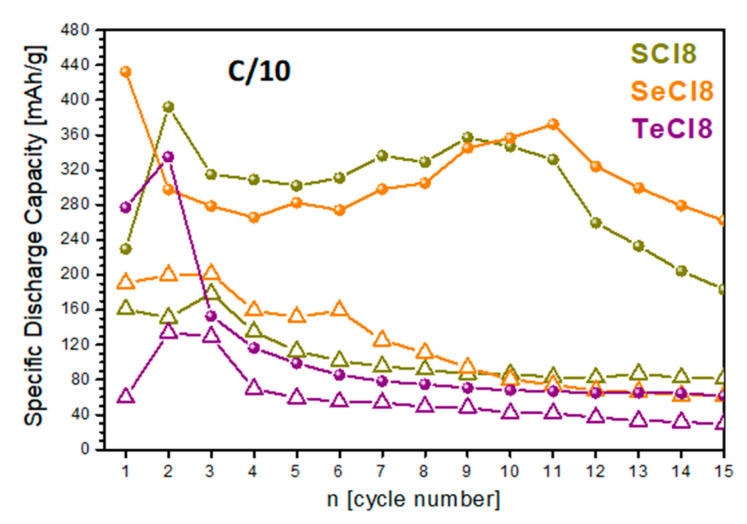
Cycling stability of **SCl8**, **SeCl8**, and **TeCl8** used as electrodes in lithium half-cells starting with a discharge mode, (—△—) pristine compounds, (—●—) composites with rGO.

Figure 7 shows the cycling stability of net NIs and their composites with rGO. First, we used low charge rates to assess whether the NI derivatives were stable during cycling. In the whole potential window (i.e., between 1.0 and 4.4 V), where both the reduction and oxidation are activated, the charge–discharge curves adopt almost the same profiles, independent of the chalcogenide nature (Figure S7A). Among the net compounds, **SCl8** delivers the highest capacity. After 15 cycles, the capacity decreases following the order **SCl8** > **SeCl8** > **TeCl8**. In synchrony with the specific capacity, the cycling stability also decreases following the same order. Dramatic improvement in the specific capacity is achieved after the addition of rGO to the net compounds (Figure 7): after 15 cycles, the discharge capacity increases from 82 to 183 mAh/g for **SCl8**; from 61 to 252 mAh/g for **SeCl8**; and from 29 to 62 mAh/g for **TeCl8**. This is directly related to the enhanced electron conductivity of NI derivatives by the addition of rGO. (It is worth recalling that rGO has an excellent electron conductivity of 7.98 × 10^−2^ Ω.cm [46].) The addition of rGO to the NI derivatives improves their capacity without altering the charge/discharge curve profiles (Figure S7B).

The curve profiles are also insensitive to whether the cell starts with charging or discharging, which is in good agreement with the CV experiments (Figure S3). Moreover, after 15 cycles, the discharge capacity is almost the same, regardless of the initial test conditions: for **SCl8**, the discharge capacity reaches 180 mAh/g compared to 183 mAh/g starting from charge and discharge, respectively.

**Figure 8:**
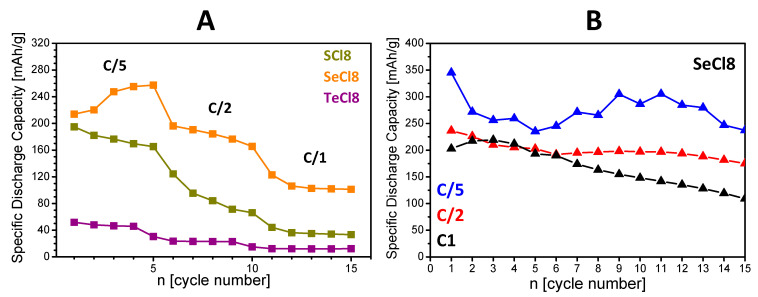
Rate capability (**A**) and cycling stability (**B**) of the composites SCl8/rGO, SeCl8/rGO, and TeCl8/rGO used as electrodes in lithium half-cells starting with a discharge mode at rates C/5, C/2, and 1C.

The next parameter revealing the storage performance of composites is the rate capability (Figure 8). The comparison reveals that the **SeCl8**/rGO composite outperforms the **SCl8**/rGO and **TeCl8**/rGO analogues. Irrespective of the used rate, the discharge capacity increases in the order **TeCl8**/rGO < **SCl8**/rGO < **SeCl8**/rGO. Importantly, at a rate of 1C, the **SeCl8**/rGO composite delivers a capacity of about 110 mAh/g, which is quite acceptable for practical applications. For this composite, the discharge capacity after 15 cycles at a rate of C/5, C/2, and 1C is 237, 204, and 193 mAh/g, respectively. These data are a good basis for further improvement of the cycling stability of **SeCl8**/rGO. This can be achieved by using an electrolyte with an optimized composition.

Ex situ SEM and EDS analyses were carried out to evaluate the composition stability of NIs after the electrochemical reaction (Figure S8). As one can see, the electrode surface remains nearly unchanged after the electrochemical test. The ratios of halogen to chalcogen atoms for **SeCl8** and **TeCl8** are as follows: Cl/Se = 1.0 in the pristine electrode versus Cl/Se = 0.7 in the tested electrode; and Cl/Te = 1.0 versus Cl/Te = 0.4. This indicates that there is a tendency for a partial loss of Cl atoms from the organic molecules, especially for **TeCl8**. The ratio is only calculated for **SeCl8** and **TeCl8** composites, as the availability of S in the electrolyte compromises the data for the **SCl8** composite. However, the partial loss of Cl atoms can be related to the limited stability of **SCl8** upon overlithiation (i.e., capacity higher than 243 mAh/g), which results in some destructive reactions in which the lithium ions abstract chlorine from the compound. Compared to **SeCl8**/rGO, **SCl8**/rGO losses capacity quicker, thus indicating that Cl atoms are detached from **SCl8** faster.

## 4. Conclusions

By combining experimental and theoretical tools, it is found that NI derivatives consisting of a *peri*-dichalcogenide bridge annulated to a naphthalimide core participate in both reduction and oxidation reactions when used as electrodes in lithium half-cells with ionic liquid electrolytes. The main building element in their molecular structure is the chalcogenide bridge, wherein a successive replacement of S with Se and Te occurs. The oxidation of NI derivatives takes place above 3.9 V with the active participation of the chalcogenide bridge: the lower the electronegativity of the chalcogenide atoms, the higher the oxidation potential. TFSI^−^ counter-ions provide the charge compensation during the oxidation of NI de-rivatives. Compared to oxidation, NI derivatives are reduced stepwise below 2.0 V with a total of four Li^+^. The first reduction step (two Li) is accomplished with the participation mainly of the chalcogenide bridge, while during the next step of reduction the imide fragment and naphthalene unit have an equal contribution to the charge reallocation. The chlorines take practically no part in the charge redistribution during reduction. During the reduction of NI derivatives up to four Li^+^, all molecular fragments remain in the reduced molecular structure with altered bond distances, whereas upon interacting with the next two Li^+^, degradation processes with the abstraction of chlorines occur.

NI derivatives display a satisfactory storage performance when used as organic electrodes in lithium half-cells. This is achieved by a simple procedure of mechanical mixing of NI derivatives with rGO in a centrifugal mixer. Among different chalcogenide-derived compounds, the best performance in terms of cycling stability and rate capability is observed for the composites of Se-containing NIs and rGO: the specific capacity is about 200 mAh/g and 300 mAh/g under current load of 50 mA/g and 20 mA/g, respectively. These favorable electrochemical properties could serve as a basis for identifying more suitable electrolyte compositions, thereby facilitating the enhancement of storage efficiency.

## Data Availability

All data generated or analyzed during this study are included in this article and its ESI. † Software used and code: (a) OriginPro 8.6 (64 bit) Sr3, b99 (Academic). (b) EC-Lab Software, Biologic. (c) ChemDraw 8.0.3. (d) GaussView 6.0. (e) Gaussian 16, Gaussian Inc.

## Supplementary Materials

The following supporting information can be downloaded at https://www.mdpi.com/article/10.3390/ma18092066/s1, Figure S1. ^1^H (A) and ^13^C (B) NMR spectra of TeCl8; Figure S2. SEM images of SCl8, SeCl8, and TeCl8 at different magnifications; Figure S3. CV curves after the fifth scan for SCl8, SeCl8, and TeCl8 used as electrodes in three-electrode cells started with a cathodic (blue lines) and anodic (red lines) scan; Figure S4. Optimized geometry of the first two lithiation steps of SCl (top), SeCl (middle) and TeCl (bottom). The first two structures on the left represent the two favorable geometries of the respective molecule with 2Li and the energy difference between them. The geometries of the complexes with 4Li are on the right; Figure S5. NBO charges of neutral oxidized and reduced SCl (A), SeCl (B), and TeCl (C); Figure S6. Chlorine abstraction due to overlithiation; Figure S7. Cycling stability of SCl8, SeCl8, and TeCl8 (A) and their composites with rGO (B) used as electrodes in lithium half-cells; Figure S8. SEM images of SCl8, SeCl8, and TeCl8 fresh (A) and cycled for 50 times (A’) electrodes in LiTFSI:Pyr_1,3_FSI electrolyte. Table S1. The reduction and oxidation capacitance, calculated from the CV curve for the net compounds and their composites with rGO; Table S2. Calculated averaged lengths (in Å) of key bonds in reduced, neutral, and oxidized SCl, SeCl, and TeCl (A = S, Se, Te); Table S3. Calculated charge distribution (summed charges) among the different molecular fragments: chalcogenide bridge (S/Se/Te), naphthalene carbons (C), imide chain (O=C-N-C=O) and chlorine (Cl);
