# Supplementary material for: Effect of the Peri-Annulated Dichalcogenide Bridge on the Bipolar Character of Naphthalimide Derivatives Used as Organic Electrode Materials
Source: Materials (Basel). 2025 Apr 30;18(9):2066. doi: 10.3390/ma18092066 (PMC12072909; doi:10.3390/ma18092066)
Supplement: Supplementary file 1 [file materials-18-02066-s001.zip › materials-3562825-supplementary.pdf]

Supplementary Information

Effect of the *peri*-Annulated Dichalcogenide Bridge on the Bipolar Character of Naphthalimide Derivatives Used as Organic Electrode Materials

Delyana Marinova,[1,]* Lyuben Borislavov,[1] Silva Stanchovska,[1] Konstantin Konstantinov,[2,3] Monika Mutovska,[2] Stanimir Stoyanov,[2] Yulian Zagranyarski,[2] Yanislav Danchovski,[1,2] Hristo Rasheev,[1,2] Alia Tadjer,[1,2] and Radostina Stoyanova[1]

[1]*Institute of General and Inorganic Chemistry, Bulgarian Academy of Sciences, 1113 Sofia, Bulgaria*
[2]*Faculty of Chemistry and Pharmacy, Sofia University "St. Kliment Ohridski", 1164 Sofia, Bulgaria*
[3]*Faculty of Pharmacy, Medical University of Sofia, 1000 Sofia, Bulgaria*

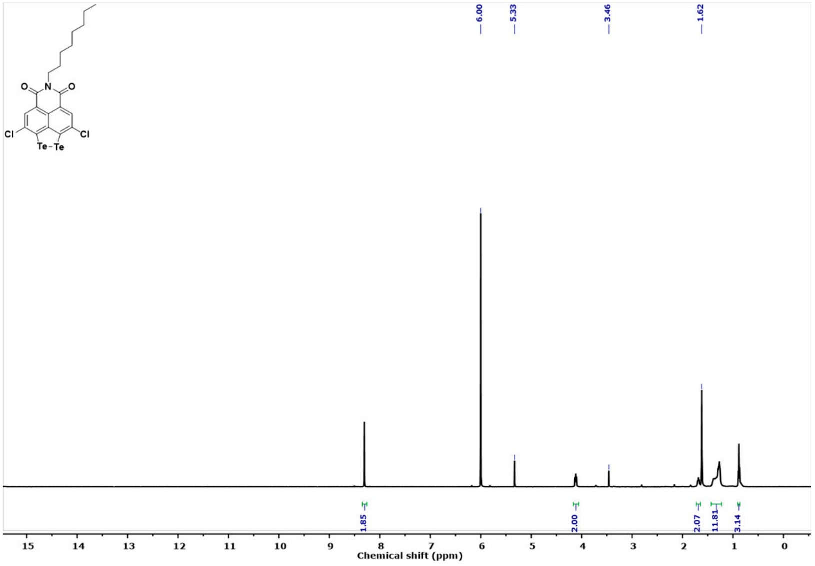

A

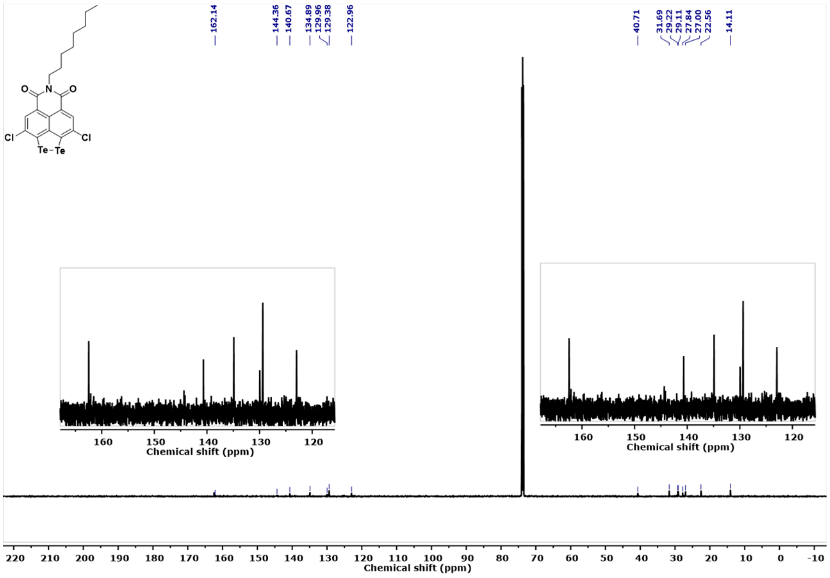

B

Figure S1. ^{1}H (A) and ^{13}C (B) NMR spectra of **TeCl8**.

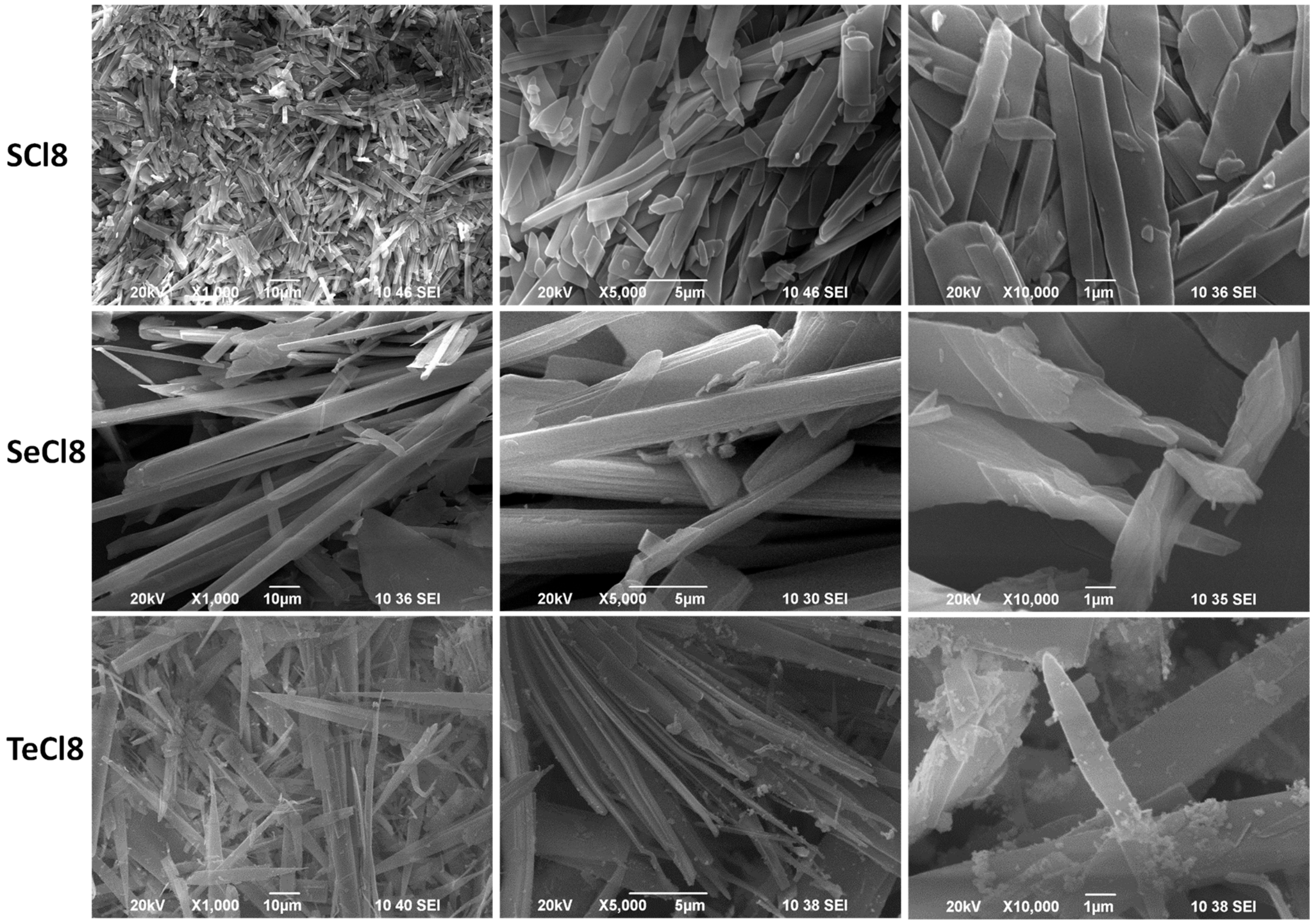

Figure S2. SEM images of **SCl8**, **SeCl8** and **TeCl8** at different magnifications.

Table S1. The reduction and oxidation capacitance (the data are calculated as specific capacity), calculated from the CV curve for the net compounds and their composites with rGO.

	Reduction capacity [mAh/g]	
	Net compounds	Composites with rGO
SCl8	132.72	159.28
SeCl8	74.30	163.83
TeCl8	41.08	68.45

	Oxidation capacity [mAh/g]	
	Net compounds	Composites with rGO
SCl8	47.26	63.98
SeCl8	22.97	49.42
TeCl8	35.36	46.38

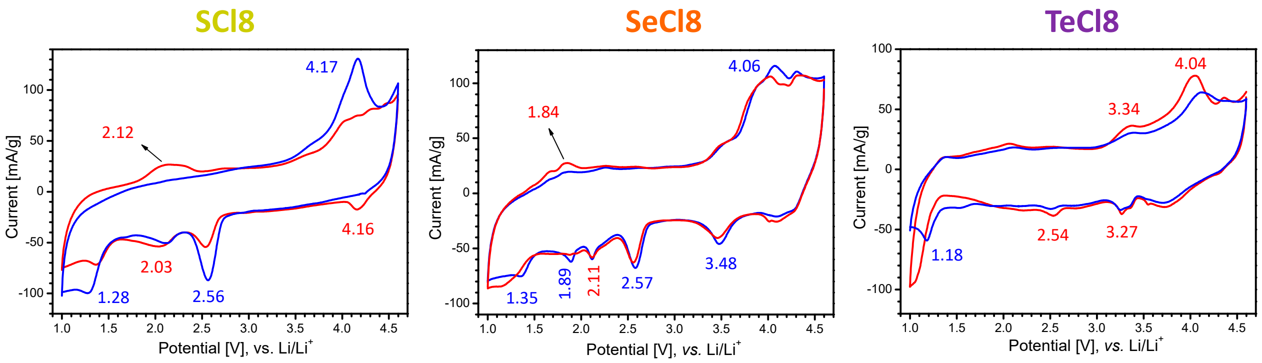

Figure S3. CV curves after the fifth scan for **SCl8**, **SeCl8** and **TeCl8** used as electrodes in three-electrode cells started with a cathodic (blue lines) and anodic (red lines) scan.

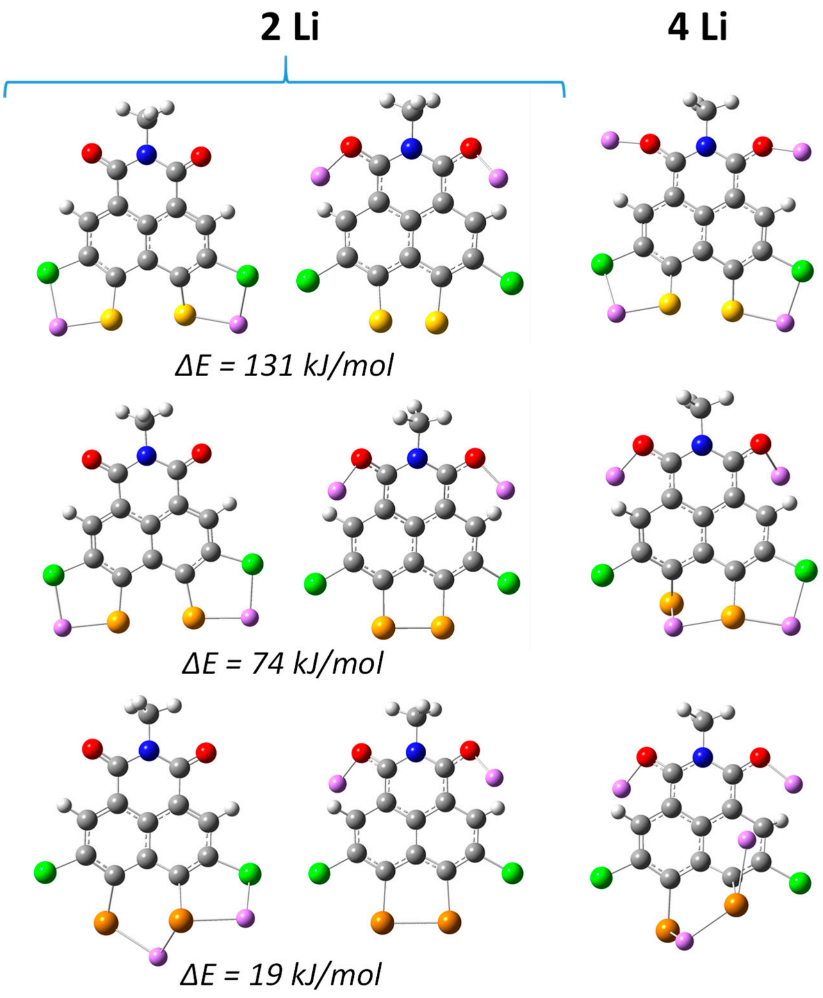

Figure S4. Optimised geometry of the first two lithiation steps of SCl (top), SeCl (middle) and TeCl (bottom). The first two structures on the left represent the two favourable geometries of the respective molecule with 2Li and the energy difference between them. The geometries of the complexes with 4 Li are on the right.

Table S2. Calculated averaged lengths (in Å) of key bonds in reduced, neutral, and oxidised SCl, SeCl, and TeCl (A = S, Se, Te)

System	(C-A)	(C-Cl)	(A-A)
SCl_+2	1.71	1.70	2.06
SCl_+1	1.73	1.73	2.09
**SCl_0Li**	**1.76**	**1.75**	**2.14**
SCl_2Li	1.75	1.80	3.16
SCl_4Li	1.77	1.87	3.19
SCl_6Li	1.77	1.88	3.26
SeCl_+2	1.84	1.71	2.30
SeCl_+1	1.86	1.73	2.33
**SeCl_0Li**	**1.88**	**1.75**	**2.39**
SeCl_2Li	1.89	1.81	3.14
SeCl_4Li	1.93	1.80	3.66
SeCl_6Li	1.95	1.77	3.72
TeCl_+2	2.08	1.72	2.67
TeCl_+1	2.11	1.74	2.71
**TeCl_0Li**	**2.13**	**1.76**	**2.77**
TeCl_2Li	2.17	1.78	3.95
TeCl_4Li	2.18	1.78	4.18
TeCl_6Li	2.18	1.79	4.00

Table S3. Calculated charge distribution (summed charges) among the different molecular fragments: chalcogenide bridge (S/Se/Te), naphthalene carbons (C), imide chain (O=C-N-C=O) and chlorine (Cl)

	**S**					**Se**					**Te**				
	**+2**	**+1**	**0**	**2**	**4**	**+2**	**+1**	**0**	**2**	**4**	**+2**	**+1**	**0**	**2**	**4**
**C(aromatic)**	-0.557	-0.870	-1.127	-1.169	-1.863	-0.746	-1.024	-1.242	-1.244	-2.055	-0.987	-1.194	-1.347	-1.282	-2.383
**S/Se/Te_sum**	1.300	0.982	0.579	-0.863	-1.047	1.556	1.174	0.702	-0.740	-1.074	1.933	1.427	0.812	-0.817	-0.856
**Li_sum**				1.741	3.568				1.701	3.581				1.654	3.504
**Cl_sum**	0.432	0.222	0.065	-0.186	-0.370	0.386	0.199	0.063	-0.189	-0.164	0.300	0.155	0.057	-0.076	-0.020
**imide chain**	-0.145	-0.247	-0.359	-0.362	-1.053	-0.159	-0.256	-0.364	-0.370	-1.059	-0.189	-0.281	-0.378	-0.333	-0.993

A

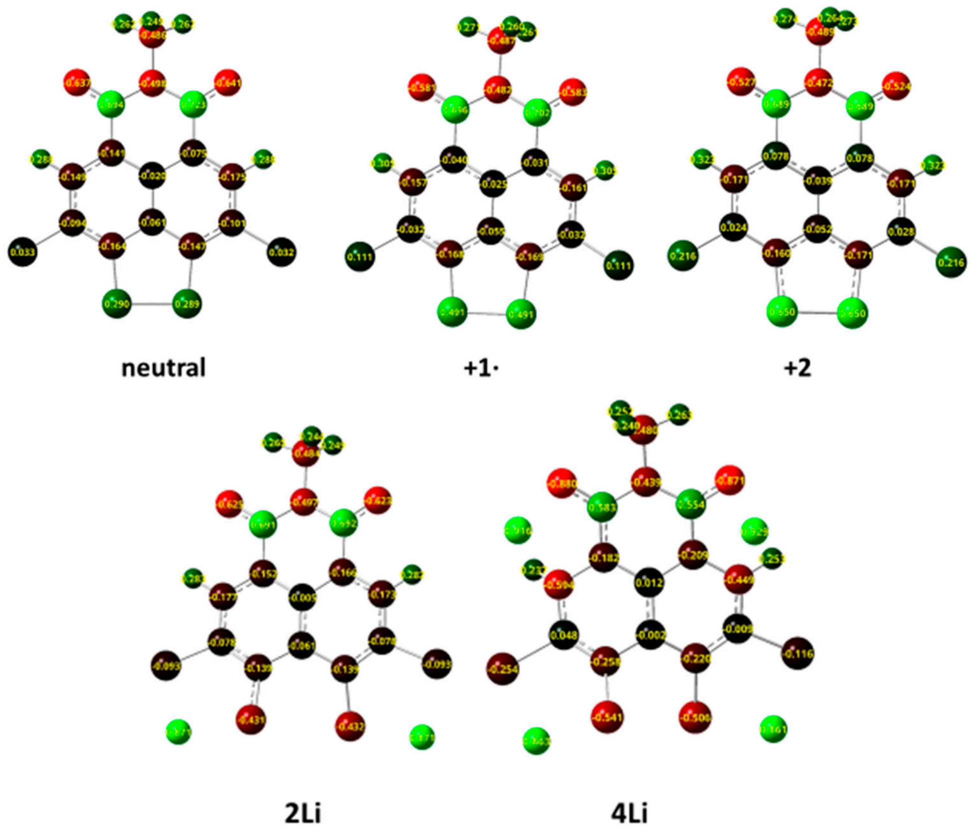

B

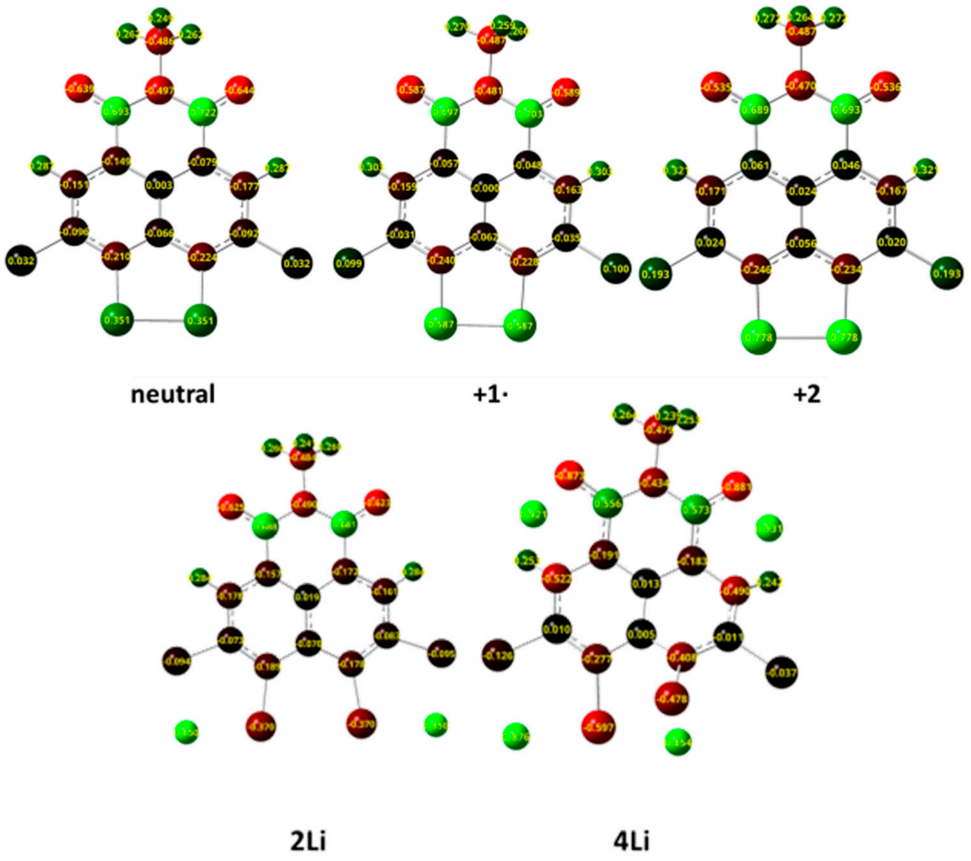

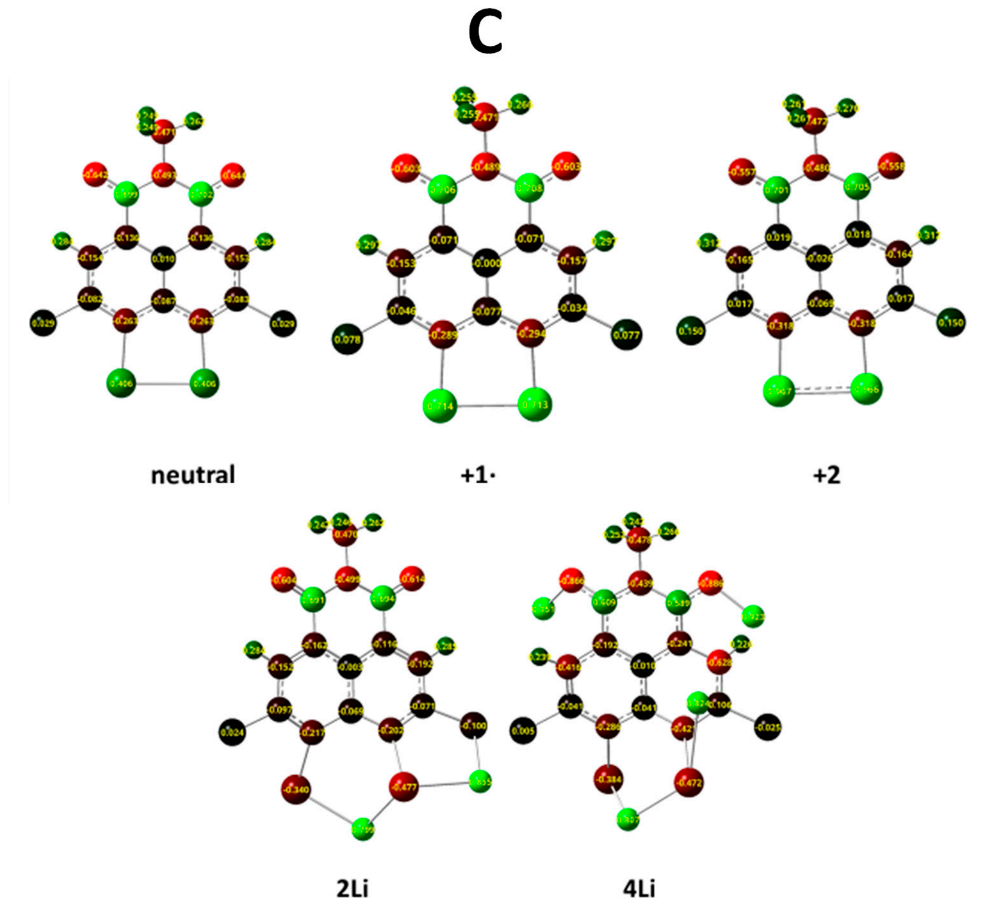

Figure S5. NBO charges of neutral oxidised and reduced SCl (A), SeCl (B), and TeCl (C)

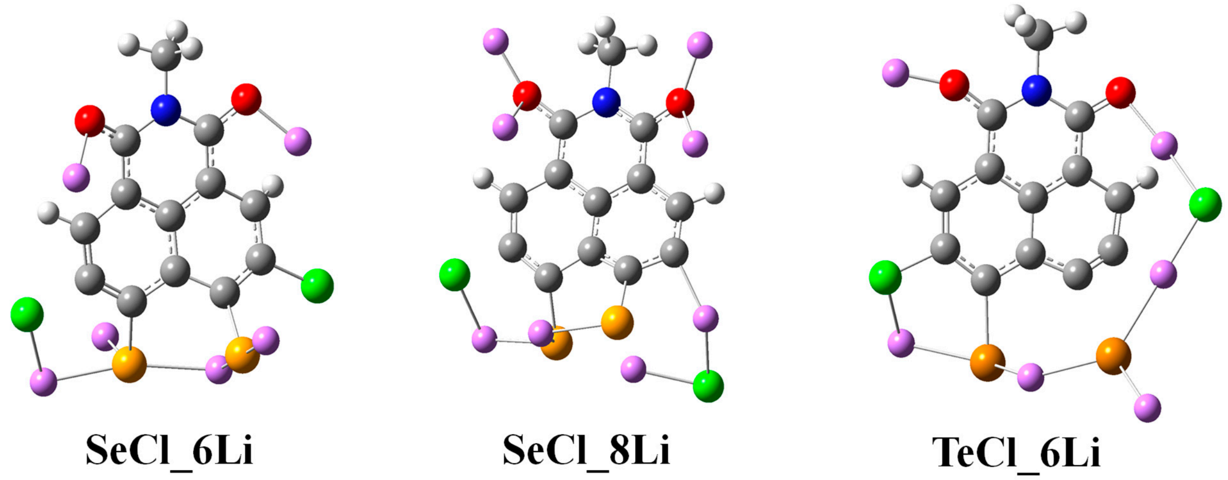

Figure S6. Chlorine abstraction due to overlithiation.

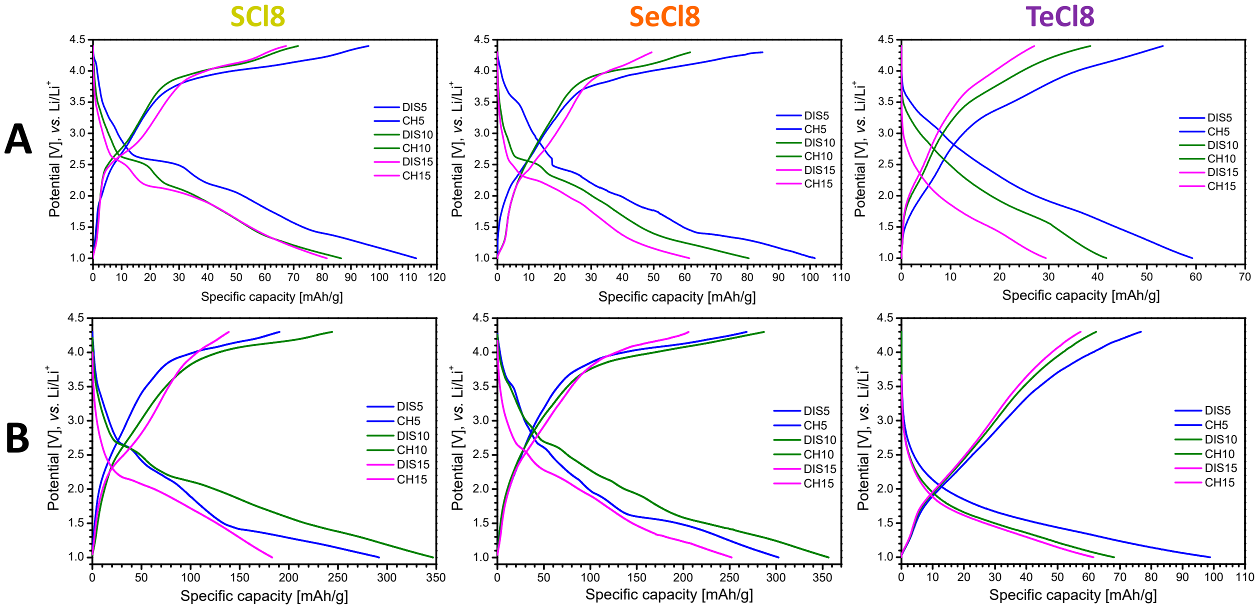

Figure S7. Cycling stability of **SCl8**, **SeCl8**, and **TeCl8** (A) and their composites with rGO (B) used as electrodes in lithium half-cells

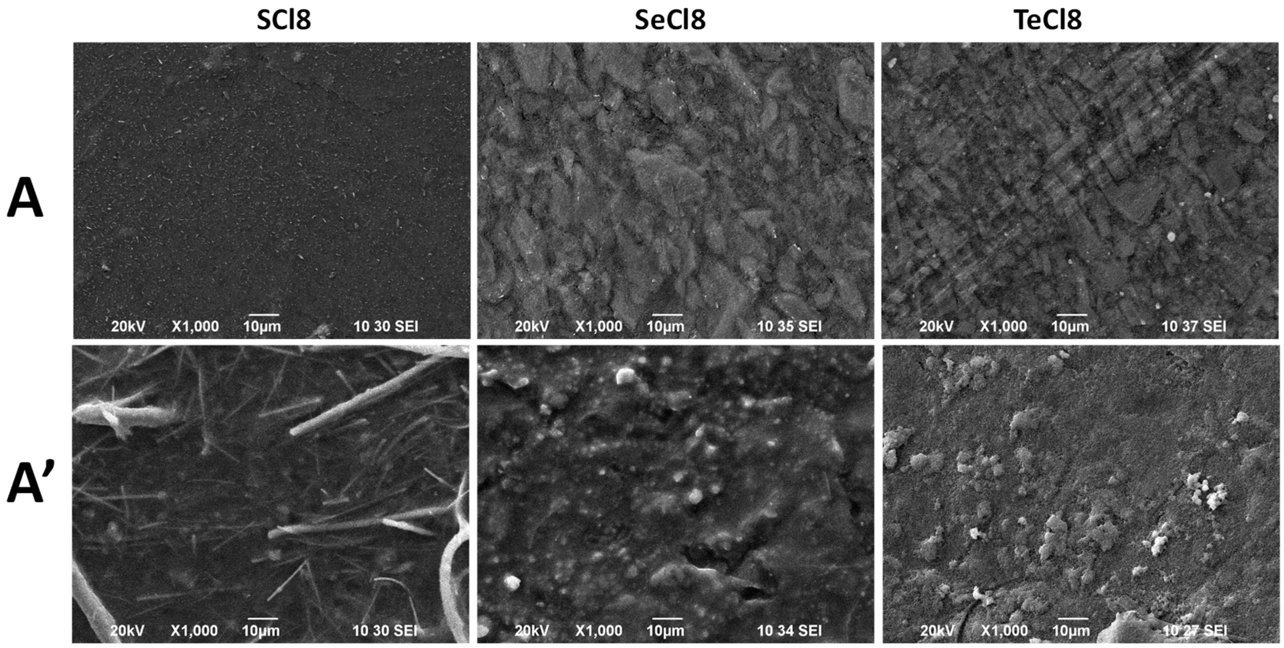

Figure S8. SEM images of **SCl8**, **SeCl8** and **TeCl8** fresh (A) and cycled for 50 times (A’) electrodes in LiTFSI:$Pyr_{1,3}$FSI electrolyte
